# The Outbreak of Small-Pox in Georgia and the Lesson It Ought to Teach

**Published:** 1882-07

**Authors:** 


### Editorial.

### THE OUTBREAK OF SMALL POX IN GEORGIA AND THE LESSON IT OUGHT TO TEACH.

In another department we publish a most interesting and important contribution toward the history of the outbreak of small-pox in this city during the present year.

The evidence, establishing the certain and inestimable value of vaccination, which is supplied by the article in question, is not new to medical men. Physicians have long been familiar with the fact, which has been tested and verified on a far larger scale, time and time again, for the last three-quarters of a century. But the testimony of a close, candid and competent observer, who is not a professional man, and who bases his conclusions upon what he has seen with his own eyes and what he has heard with his own ears, uninfluenced by early training and unbiased by preconceived opinions, must, we feel confident, carry conviction to the minds of many who doubt, strengthen the faith of many who are weak and awaken the interest of many who are indifferent. It is with this hope that we urge attention to the recital of events which have transpired in our own midst and at our very doors, as calculated to produce impressions beyond the reach of happenings and arguments more remote and less intimately associated with the daily lives of those who are most nearly concerned.

Who can question the probability of serious consequences if the protective power of the simple, yet sufficient, prophylactic had not been appealed to? Who can estimate the damaging results to this city and to this state if the health authorities of Atlanta had not responded with an earnest and determined zeal when the very first signal of danger appeared? Who can say how extensive and how violent an epidemic might have been fanned into alarming proportions by a little delay or a little neglect on the part of the Atlanta Board of Health when the dread invader first appeared in her midst?

These questions faintly suggest what disaster has been averted and what suffering has been avoided by the prompt, intelligent and unflinching interposition of the means at the command of Georgia's capital.

But suppose that this, or any other pestilential disease should invade the state at a point unable or unwilling to put forth the energy or to expend the treasure necessary to overpower and expel the enemy. What would be the result?

This is a question worthy of the serious attention of sanitarians, of statesmen, of business men, of farmers, and, indeed, of all classes of our people.

Our present state laws are totally inadequate, and their execution is entirely untrustworthy. The people of the state are not protected as they should be from the ravages of preventable diseases; on the contrary they often, through carelessness or ignorance, invite disease and death. As illustrative of the reckless disregard of the sanitary demands of the state we will refer to a recent act of her chief executive. The governor is required by statute to furnish vaccine virus to the ordinaries of the several counties of this state upon application. Accordingly, about two months ago, a formal demand was made upon the governor for vaccine virus by the officials of this county, and finally, as the result of repeated appeals, he actually consented to do his duty and promised to execute the law. Notwithstanding which not a particle of virus has ever been received from the state or any agent thereof. It is true the call for virus to protect the lives of the people did not offer an opportunity to tell stale jokes or to recite anecdotes of questionable propriety at a Sunday school mass meeting; neither did it involve the necessity of delivering a stump speech to a devout congregation of negro voters. The request only demanded a quiet and conscientious discharge of a sacred duty, and, under the circumstances, perhaps it was unreasonable to expect success. Certain it is, success was not achieved, and it was left for the city and the county to provide, as best they could, the means for meeting the emergency, and thereby shielding the state from a formidable invasion.

Now, what is the moral of all this? What practical lesson does it teach? To our mind the answer is plain. It indicates, unquestionably, the necessity for a capable, discreet and well equipped State Board of Health.

No civilized community can afford to dispense with the services of such a safeguard, and the expense incident to the proper maintenance of a state health department is of trifling consequence in comparison with the benefits which will inevitably ensue from the establishment of a properly organized sanitary council.

Our space will not permit, at present, an elaboration of this idea, but the urgent demand for such an organization has been brought home to many of our citizens by recent experiences, and we commend the simple suggestion to the thoughtful consideration of our readers.

